# First Ecuadorian Pediatric Case of Multisystem and Neurological Involvement Associated with Influenza A—H5N1 Virus—Case Report

**DOI:** 10.3390/v18070749

**Published:** 2026-07-07

**Authors:** Frances Fuenmayor, Santiago Chávez, María de los Ángeles Costta, Mateo Carvajal, Denisse Benítez, Rommel Guevara, Erika Muñoz, Paúl Cárdenas, Marisol Carrillo, Marcelo Guerrero, Melanie Orellana

**Affiliations:** 1 Baca Ortiz Pediatric Hospital, Quito 170523, Ecuador; macosttamichuy@hotmail.com; 2 School of Medicine, Universidad de las Américas, Quito 170124, Ecuador; santiago.chavez.fuenmayor@udla.edu.ec (S.C.); melanie.orellana@udla.edu.ec (M.O.); 3 Institute of Microbiology, COCIBA, Universidad San Francisco de Quito, Quito 170901, Ecuador; mcarvajal@usfq.edu.ec (M.C.); dbenitezq@usfq.edu.ec (D.B.); rguevara@estud.usfq.edu.ec (R.G.); emunoz@usfq.edu.ec (E.M.); pacardenas@usfq.edu.ec (P.C.); 4 Intensive Care Medicine, Pontifical Catholic University of Ecuador, Quito 170525, Ecuador; medicsol@outlook.com (M.C.); mguerrero509@puce.edu.ec (M.G.)

**Keywords:** viral encephalitis, influenza A H5N1, pediatrics, neurological complications, oseltamivir, case report

## Abstract

Influenza A (H5N1) is a highly pathogenic zoonotic virus with a human fatality rate of approximately 60%. Pediatric cases and associated neurological manifestations remain poorly documented in Latin America. This report describes the first confirmed Ecuadorian pediatric case of H5N1-associated encephalitis and multisystem organ failure in a previously healthy 9-year-old female following direct contact with infected poultry. The clinical course was characterized by an atypical initial presentation of bilateral periorbital edema and headache, progressing to acute encephalitis, cerebral ischemia, flaccid tetraplegia, central diabetes insipidus, and refractory septic shock. Diagnostic confirmation was achieved via nasopharyngeal RT-PCR, with additional RT-PCR and sequencing performed on cerebrospinal fluid, which identified conserved influenza A M1/M2 gene fragments, while laboratory markers—including marked elevations in IL-6, ferritin, and CRP—indicated a severe hyperinflammatory state. Management involved an intensive multidisciplinary approach utilizing oseltamivir, intravenous immunoglobulin, modulated-dose corticosteroids, desmopressin, and mechanical ventilation. Despite a severe clinical course, the patient achieved a favorable recovery, with a Glasgow Coma Scale score of 15/15 at discharge and only partial residual paresis and left hypoacusia as sequelae. This landmark case provides rare evidence of H5N1 neuroinvasion in a pediatric patient and demonstrates that timely detection combined with aggressive immunotherapy and antiviral treatment can improve survival. Furthermore, it underscores the critical necessity for strengthened regional molecular surveillance and clinical training to recognize atypical presentations of emerging zoonoses in Latin America, especially in cases involving contact with sick poultry.

## 1. Introduction

Since its identification in Hong Kong in 1997, influenza A (H5N1) has been responsible for multiple sporadic outbreaks, with an overall case fatality rate of approximately 60% [1,2]. Although human-to-human transmission remains limited, its high virulence and potential for genetic adaptation have raised global concerns about the possibility of a future pandemic [3,4]. Recent literature has documented a progressive increase in human cases with neurological manifestations associated with H5N1, suggesting marked neurotropism of the virus, particularly in pediatric patients [5,6].

In children, influenza A (H5N1) infections are rare, but when they occur, they often present with atypical, rapidly progressing clinical manifestations, frequently with multisystem involvement, neurological dysfunction, and high mortality [6,7]. Diagnosis is particularly difficult in regions with a high background of respiratory infections and limited zoonotic surveillance, where disease recognition and timely treatment are often delayed. Recent studies have emphasized the importance of including avian influenza in the differential diagnosis of encephalitis and febrile illnesses of undetermined etiology in areas with a history of avian mortality or exposure to infected animals [8,9].

This case represents a historic milestone in pediatric medicine in Ecuador. More than its clinical significance, it highlights biological resilience and medical advancement. It shows that despite a highly lethal viral strain, prompt intervention, teamwork across disciplines, and diligent epidemiological surveillance can prevent death. The case not only questions global infant mortality statistics for influenza A (H5N1) but also underscores the urgent need to enhance molecular diagnostic systems, pediatric intensive care units, and medical training in response to emerging zoonoses.

## 2. Clinical Case Presentation

A 9-year-old girl arrived at Ambato Regional Hospital exhibiting coryza, conjunctival pruritus, symmetric bilateral periorbital edema (without proptosis or visual impairment), diffuse abdominal pain, a severe headache, and a fever that had started approximately eight days earlier. A timeline summary is provided in Table 1.

**Figure 1:**
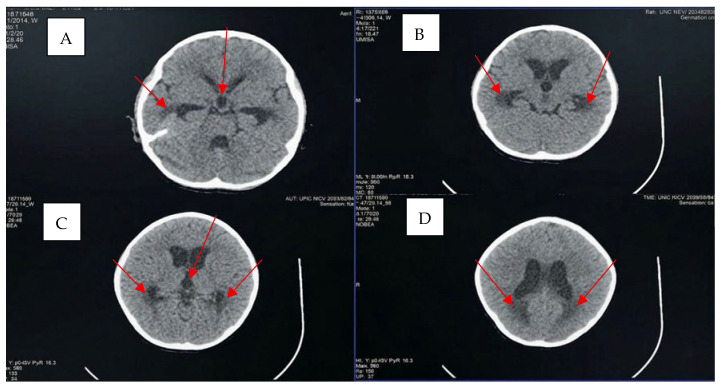
Non-contrast axial head computed tomography (CT). Red arrows indicate the identified pathological abnormalities, specifically the heterogeneous hypodense lesions and focal ventricular dilatations. (**A**) Bilateral hypodense regions with heterogeneous attenuation and irregular margins are identified lateral to the floor of the anterior temporal horns. An additional heterogeneous hypodensity with irregular margins is noted along the anteromedial floor of the left frontal horn. Focal saccular dilatation of the posterior aspect of the third ventricle is also present. (**B**) Bilateral hypodense regions are noted lateral to the bodies of the temporal horns; the margins of these areas conform to the convexity of the adjacent ventricular structures. Saccular dilatation of the mid-portion of the third ventricle is evident. (**C**) Bilateral heterogeneous hypodensities with irregular margins are located lateral to the ventricular atria, accompanied by focal saccular dilatation of the anterior aspect of the third ventricle. (**D**) Bilateral heterogeneous hypodensities with irregular margins are identified posterior to the central portions of the frontal horns.

**Figure 2:**
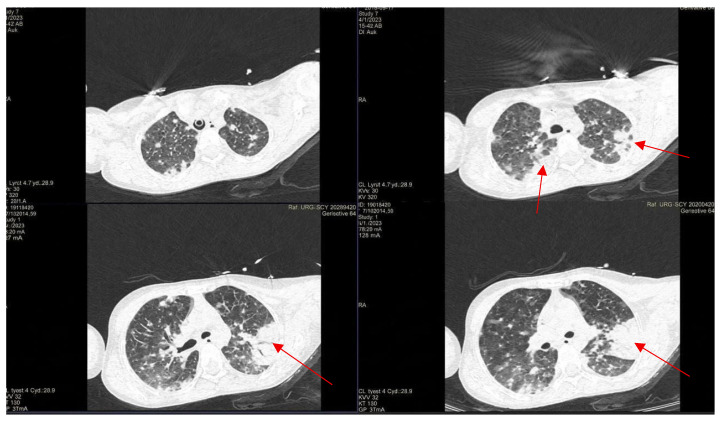
Non-contrast chest computed tomography (CT), axial view, lung window. The images demonstrate consolidated lesions in the right upper, left upper, and lower lobes. Additionally, multiple diffusely distributed foci of nodular consolidation are present throughout both pulmonary hemifields. Red arrows highlight the prominent areas of consolidation.

Initially, her parents managed her early condition with home remedies. She had no prior medical history, no chronic illness, and no immunosuppressive therapy. Her routine childhood immunizations (Ecuadorian national schedule, including BCG, pentavalent, MMR, varicella, and yellow fever) were complete and age-appropriate; she had not received seasonal influenza vaccination in the preceding 12 months and had never received any H5-specific or pre-pandemic influenza vaccine. On directed questioning at Baca Ortiz Pediatric Hospital (HPBO) admission, the family reported direct handling of dead backyard poultry approximately 72 h before symptom onset, in a rural setting with documented avian mortality in the local flock. This zoonotic exposure was the pivotal element that prompted clinicians to broaden the differential beyond bacterial meningitis and community-acquired pneumonia to include highly pathogenic avian influenza, to escalate biosafety precautions to airborne and contact isolation with N95 use, to notify the National Institute of Public Health Research (INSPI), and to prioritize nasopharyngeal sampling for influenza A subtyping by RT-PCR alongside the empirical antibiotic and acyclovir coverage already in place. When her condition worsened, she was brought to Ambato Regional Hospital, where she was admitted and treated emergently. Twenty-four hours after admission, her condition deteriorated further, with respiratory distress and altered mental status. Based on initial evaluations and suspicion of bacterial meningitis, a CT scan (see Figure 1 and Figure 2) and cerebrospinal fluid analysis (see Table 2) were performed. Referral protocols to our facility, HPBO, Quito, Ecuador, were activated. At HPBO, the initial exam revealed three key findings: bilateral periorbital edema, a temperature above 39 °C, and severe headache. Subsequent labs and imaging studies, including brain and chest CT scans and a brain MRI, were ordered.

Day 1—Admission and initial evaluation. The following are the relevant semiological findings from the patient’s first day of admission to HPBO: In the neurological area, the patient presented a Glasgow Coma Scale (GCS) score of 3T/15 (RASS−5). Protocols for deep analgosedation and invasive mechanical ventilation were initiated, and there was a complete absence of response to both verbal and painful stimuli. In the cardiovascular area, volume-refractory septic shock was confirmed, requiring noradrenaline as vasoactive support. In the respiratory area, a mechanical ventilation protocol with protective parameters was implemented, and an anteroposterior (AP) chest X-ray showed bilateral pulmonary consolidations suggestive of pneumonia of undetermined etiology (see Figure 2). In the metabolic area, severe hypernatremia was observed at 166 mEq/L, along with elevated serum osmolarity (>300 mOsm/kg) and low urinary osmolarity (<300 mOsm/kg), a constellation diagnostic of central diabetes insipidus. Laboratory studies on admission revealed marked leukocytosis (19.3 × 10^3^/µL) with neutrophilia (93%), C-reactive protein 5.13 mg/dL, procalcitonin 24.3 ng/mL, ferritin 1301 ng/mL, and interleukin-6 25.2 pg/mL—a profile consistent with severe sepsis superimposed on a hyperinflammatory state suggestive of macrophage activation. Adrenocortical evaluation showed a low morning cortisol of 2.20 µg/dL, raising concern for transient panhypopituitarism secondary to hypothalamic involvement. Coagulation studies, hepatic transaminases, lactate, and arterial blood gases were monitored serially throughout the intensive care course; no overt disseminated intravascular coagulation was documented. Cerebrospinal fluid examination yielded cloudy fluid with glucose 32 mg/dL, elevated protein, and 153 white blood cells/µL (6% polymorphonuclear), compatible with a meningoencephalitic process of viral or inflammatory origin. The integrated laboratory profile therefore framed three concurrent processes: a severe systemic inflammatory response, a meningoencephalitic CNS process, and an evolving neuroendocrine dysfunction.

On Day 2, there was persistence of septic shock criteria. A rapid influenza antigen test on a nasopharyngeal swab returned positive for influenza A; the result was subsequently confirmed by RT-PCR (subtyped as H5N1) on the same nasopharyngeal sample. On neurological reassessment, the patient presented with disorientation and a Glasgow Coma Scale score of 8/15. The clinical picture was compatible with encephalitis, as evidenced by tetraplegia (Daniels Muscle Strength Scale score 0/5 in all four limbs) and diffuse restriction on brain MRI, as shown on ADC and DWI sequences. These findings suggest direct vascular endothelial damage, probably associated with the inflammatory process.

**Figure 3:**
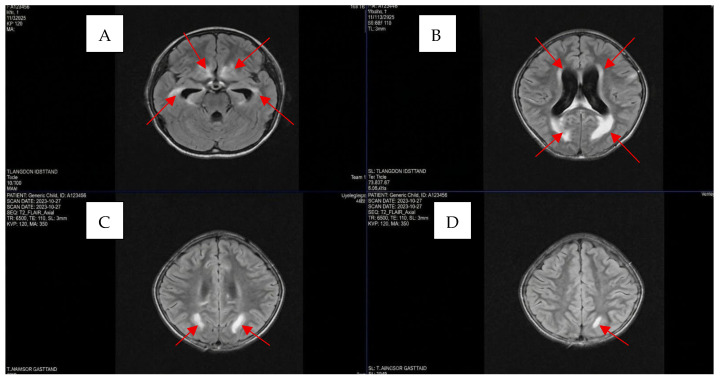
Non-contrast axial T2-FLAIR magnetic resonance imaging of the brain. Red arrows indicate the identified hyperintense lesions across the specified regions. (**A**) Bilateral heterogeneous hyperintensities located laterally to the ventral temporal horns, in the medial-dorsal floor of the frontal lobes, and at the junction of the optic chiasm. (**B**) Bilateral periventricular heterogeneous hyperintensities, along with posterior homogeneous hyperintensities displaying an arciform pattern in the parieto-occipital subcortical white matter (sparing the U-fibers). (**C**) Bilateral heterogeneous hyperintense lesions within the parietal subcortical white matter. (**D**) A solitary homogeneous hyperintense focus in the left parietal subcortical white matter, similarly sparing the U-fibers.

**Figure 4:**
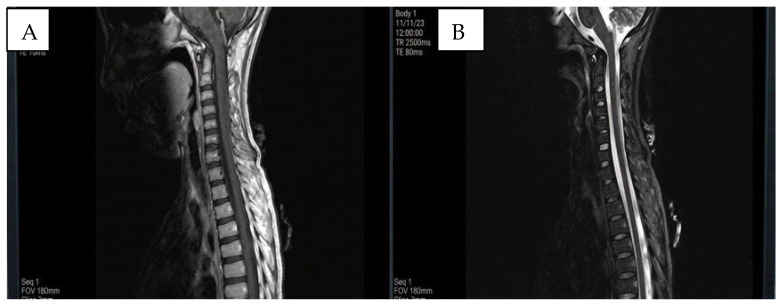
Non-contrast sagittal magnetic resonance imaging (MRI) of the cervicothoracic spine. (**A**) T1-weighted sequence demonstrating preserved vertebral bodies and no significant signal intensity alterations along the spinal cord. No obvious musculoskeletal abnormalities are noted. (**B**) T2-weighted sequence confirming the absence of pathological signal changes within the spinal cord path, with preserved surrounding musculoskeletal structures.

Day 3—Definitive imaging characterization and antiviral initiation. The findings on non-contrast MRI of the brain and spinal cord are presented (Figure 3 and Figure 4). Hyperintense foci with diffuse characteristics and heterogeneous borders were visualized in FLAIR/T2 sequences, mainly in the periventricular and parieto-occipital regions. These lesions suggested a broader differential than initially considered. The leading diagnostic possibilities were: (i) viral encephalitis with a neurotropic pathogen (with H5N1 favored given the zoonotic exposure, but herpes simplex virus, varicella-zoster virus, enteroviruses, and arboviruses were also considered until excluded by CSF PCR); (ii) acute disseminated encephalomyelitis (ADEM) given the multifocal subcortical white-matter involvement; (iii) bacterial meningoencephalitis with secondary cerebrovascular complications, supported initially by CSF pleocytosis and elevated PCT; (iv) hypoxic-ischemic injury secondary to refractory septic shock; and (v) a hyperinflammatory or cytokine-storm-mediated endothelial injury, supported by the marked elevations in IL-6, ferritin, and CRP. The combined imaging pattern—bilateral parieto-occipital subcortical hyperintensities, caudate involvement, and diffuse restriction on DWI/ADC—ultimately favored a mixed encephalitic and ischemic process, consistent with H5N1 neuroinvasion combined with cytokine-driven endothelial damage (see Table 3). Following review of the Day-2 rapid antigen result and confirmation by RT-PCR on the morning of Day 3, oseltamivir was initiated at a dose of 75 mg orally every 12 h (weight-adjusted: 4.5 mg/kg/dose) for 10 consecutive days. The brief delay between the positive rapid test and treatment initiation reflected the institutional protocol requiring molecular confirmation prior to starting weight-adjusted antiviral therapy in a critically ill pediatric patient already on broad empirical coverage.

Days 5–23—Clinical evolution, immunomodulation, and recovery. On Day 5, analgosedation was weaned. On reassessment, the Glasgow Coma Scale score was 6T/15. Tetraplegia persisted, with muscle strength of 0/5 in all four limbs, and decreased osteotendinous reflexes were observed in all four extremities (1/4). On Day 7, upon reassessment of respiratory mechanics, clinical improvement was observed, allowing for successful extubation. The Glasgow Coma Scale score improved to 8/15; however, tetraplegia persisted. Laboratory confirmation of influenza A—H5N1 was obtained.

On Day 8, a cerebrospinal fluid (CSF) sample was obtained for viral diagnosis and RT-PCR identification. The National Institute of Public Health Research (INSPI) confirmed the diagnosis of H5N1. For complementary viral genome sequencing, an aliquot of the CSF sample was sent to the Institute of Microbiology at the Universidad San Francisco de Quito. The informed consent signed by the patient’s parents includes the sample’s sequencing and storage. At USFQ, the positivity of the sample was confirmed by quantitative reverse transcription PCR (RT-PCR) using the Veri-Q PCR Kit (MiCo BioMed, Seongnam-si, Republic of Korea), targeting the M gene. For viral sequencing, two library preparation methods were used: one based on total nucleic acid (DNA and RNA) and another using the SISPA amplification strategy to enrich viral sequences. Sequencing was performed using GridION Oxford Nanopore Technologies protocols (Oxford, UK): SQK-LSK112, a ligation-based kit designed for high-precision long reads, and SQK-PBK004, a PCR barcode kit commonly used for fragmented or low-input material. Four datasets corresponding to different library preparations were obtained. Quality assessment of the reads using NanoPlot revealed predominantly short reads, mostly below 2000 bases, with moderate Phred quality scores ranging between 9 and 15. Although some reads exceeded 5000 base pairs and a few approached 9000 base pairs, the overall data were fragmented and showed limited accuracy for whole genome assembly. The resulting FASTQ files were initially analyzed using the Epi2Me wf-flu workflow; however, no positive results were obtained due to insufficient genomic coverage, which prevented influenza typing and clade classification. A second analysis using the wf-metagenomics workflow was performed, converting reads to FASTA format and aligning them against a custom BLAST (v. 2.16.0) database constructed from the Orthomyxoviridae family. This analysis revealed short sequence fragments (28–41 base pairs) mapping to the conserved M1/M2 genes of the influenza A virus. These genes are common to all influenza A strains, confirming viral presence but lacking sufficient resolution for subtype or clade identification. Although bacterial sequences were also detected, likely reflecting coinfection in a hospitalized patient, they were excluded from interpretation to focus on viral findings. Overall, the data confirmed the presence of influenza A through conserved gene matches but did not support deeper genomic characterization.

On Days 10–11 of hospitalization (counted from admission to HPBO), intravenous immunoglobulin (IVIG) therapy was administered at 1 g/kg/day for 2 consecutive days. On re-evaluation with a head CT scan, diffuse hypodense lesions were observed in the subcortical white matter, particularly in the parieto-occipital regions. On Days 20–23, the Glasgow Coma Scale score improved to 9/15. Reassessment using the Daniels Muscle Strength Scale showed progressive improvement in tetraplegia, with a score of 2/5. Follow-up MRI demonstrated lesions consistent with subacute ischemic changes in both caudate nuclei and the bilateral parieto-occipital subcortical white matter. On Day 23, the patient was discharged from the hospital. Prior to discharge, the following findings were recorded: a Glasgow Coma Scale score of 15/15, partial residual paresis, and left hypoacusis. No healthcare-associated infections were ever documented in this patient. All days of IVIG treatment described in this manuscript refer specifically to the days elapsed since admission to the Baca Ortiz Pediatric Hospital (HPBO) (please refer to Table 4 and Table 5 for detailed information on interventions, therapy, and support.).

## 3. Discussion

The atypical initial presentation—nonspecific symptoms such as eyelid edema, headache, and abdominal pain, without overt pneumonia—delayed the suspicion of a severe viral infection. This nonspecific pattern is consistent with the findings of Nakajima et al. (2022) [7], who reported that pediatric patients with H5N1 often present with non-respiratory initial symptoms that complicate the differential diagnosis with bacterial infection. The limited availability of rapid molecular diagnostics in secondary-level hospitals further delays etiological confirmation and the timely initiation of antiviral therapy [9].

The neurotropism of H5N1 and its capacity to cross the blood–brain barrier [1,10,11] produce severe neurological manifestations that can mimic other infectious or autoimmune etiologies [12], increasing the risk of underdiagnosis. In our case, the syndromic presentation of tetraplegia, ischemic lesions, and central diabetes insipidus posed a unique diagnostic challenge, as these findings are rarely directly associated with the influenza A—H5N1 virus. RT-PCR confirmation of nasopharyngeal swabs, along with supplementary cerebrospinal fluid results, was decisive, reinforcing the need to consider H5N1 within the etiological spectrum of viral encephalitis in patients with a history of avian exposure. Overall, this case illustrates how the absence of a classic respiratory presentation, the similarity to bacterial infections, and the limited pediatric literature on viral neuroinvasion hindered our early diagnosis.

Recent literature reports that mortality is high among cases with central nervous system involvement, with survival rates of 40–60%. Key factors include the time from symptom onset to the initiation of antiviral treatment and the extent of multi-organ involvement [1,3]. Survivors often present with persistent neurological sequelae, such as paresis, cognitive impairment, post-encephalitic epilepsy, or endocrine dysfunction due to hypothalamic–pituitary damage (as evidenced in the presented clinical case). Recent studies, including those by Zhang et al. (2022) [5] and Bauer et al. (2023) [6], have confirmed that direct neuronal damage mediated by neuroinvasion and cytokine storm exacerbates cerebral edema and multifocal ischemia, leading to a high risk of permanent neurological disability even after resolution of the infectious condition. Given the simultaneous administration of multiple therapeutic agents—including oseltamivir, antibiotics, acyclovir, corticosteroids, IVIG, desmopressin, mechanical ventilation, and vasopressors—it is not possible to attribute clinical improvement to any single intervention.

This case underscores the severity of multisystemic involvement in influenza A H5N1 in pediatric patients, as well as the importance of a structured follow-up protocol for managing neurological and metabolic sequelae. Our findings highlight the need for active surveillance in children exposed to avian-influenza risk factors and for protocols covering early identification, continuous monitoring, and rehabilitation after resolution of the acute illness. Given the magnitude of these clinical implications, primary prevention through zoonotic control remains a fundamental pillar for reducing the incidence of new cases.

## Figures and Tables

**Table 1:** Clinical case timeline summary.

Time Point	Clinical Events
Day −7 to −2 (Pre-admission)	Previously healthy pediatric patient developed acute fever, coryza, conjunctival pruritus, bilateral periorbital edema, diffuse abdominal pain, headache, and malaise without prominent respiratory symptoms. Managed conservatively at home.
Day −1	Progressive neurological symptoms appeared including lethargy, irritability, and altered mental status, prompting urgent medical evaluation.
Day 0 (Hospital admission)	Admission to the emergency department due to fever and acute encephalopathy. Initial laboratory evaluation revealed inflammatory markers. Neuroimaging was requested. The patient was transferred to the PICU for close monitoring.
Day 1	Neurological deterioration with seizures and decreased level of consciousness, requiring airway protection and initiation of mechanical ventilation. Empirical broad-spectrum antimicrobials and antiviral therapy were started. Lumbar puncture and extended infectious workup were performed.
Day 2–3	Brain MRI demonstrated findings consistent with acute necrotizing encephalopathy, including bilateral thalamic involvement. CSF analysis showed inflammatory changes. Molecular testing confirmed influenza A (H5N1) infection.
Day 3–5	Targeted antiviral therapy continued. Immunomodulatory treatment with intravenous immunoglobulin and corticosteroids was initiated. Hemodynamic and neurological stabilization began.
Day 6–10	Gradual neurological improvement with reduced seizure frequency and partial recovery of consciousness. Progressive weaning from mechanical ventilation was achieved.
Day 11–14	Successful extubation and transfer from PICU to the general pediatric ward. Persistent but improving neurological deficits were noted on examination.
Hospital discharge	Discharge after multidisciplinary assessment with residual neurological sequelae. A structured rehabilitation and outpatient follow-up plan was established.
Short-term follow-up	Partial neurological recovery documented during outpatient follow-up, with continued need for neurocognitive and rehabilitative support.

**Table 2:** Summary of analytical findings and data interpretation.

Parameter	Finding	Interpretation
Blood count	Leukocytosis—(19.3 × 10^3^/µL) Neutrophilia (93%)	Secondary bacterial infection/systemic inflammatory response
CRP/PCT	CRP: 5.13 mg/dL (ref. < 0.5 mg/dL; ↑) PCT: 24.3 ng/mL (ref. < 0.5 ng/mL; ↑↑)	Severe sepsis
Cerebrospinal fluid analysis	Cloudy Glucose: 32 mg/dL (↓) Proteins: ↑ White blood cells: 153/µL PMN—6%	Meningoencephalitis (viral vs. inflammatory)
Ferritin/IL-6	Ferritin: 1301 ng/mL (↑↑); IL-6: 25.2 pg/mL (↑)	Macrophage activation syndrome/hyperinflammation
Nasopharyngeal swab	Positive PCR for influenza A H5N1	Etiological confirmation
Adrenocortical hormones	Low cortisol (2.20 µg/dL)	Transient panhypopituitarism due to hypothalamic involvement

Abbreviations: CRP, C-reactive protein; PCT, procalcitonin; PMN, polymorphonuclear leukocytes; IL-6, interleukin-6; PCR, polymerase chain reaction; µL, microliter; ng/mL, nanograms per milliliter; pg/mL, picograms per milliliter; µg/dL, micrograms per deciliter. Symbols: (↑) indicates an increase relative to normal reference values; (↑↑) indicates a marked increase; (↓) indicates a decrease.

**Table 3:** Summary of imaging findings and data interpretation.

Study	Finding	Relevance
Initial Brain CT Scan	Hypodense lesions in periventricular (parieto-occipital) white matter	Compatible with vasogenic edema and encephalitis
Brain MRI (Day 3 of Hospitalization)	Hyperintensities on FLAIR/T2: caudate nuclei and parieto-occipital cortex	Subacute ischemic lesions/neuroinflammation
Chest X-ray, Anteroposterior (AP) View	Bilateral consolidation, nodular infiltrates	Severe influenza pneumonia
Brain and Spinal Cord MRI (Day 4 of Hospitalization)	Without spinal cord abnormalities	Rule out associated myelitis

Abbreviations: CT, computed tomography; MRI, magnetic resonance imaging; FLAIR, fluid-attenuated inversion recovery; AP, anteroposterior.

**Table 4:** Pharmacological interventions.

Drug/Intervention	Dosage and Administration	Duration	Power/Justification	Changes and Explanation
Oseltamivir	75 mg/12 h PO (adjusted for weight: 4.5 mg/kg/dose)	10 days (from admission)	High antiviral potency: Inhibits H5N1 neuraminidase. Initiation delayed to Day 3 pending diagnostic confirmation.	The treatment course was extended to 10 days (H5N1 protocol) due to persistent viral load in the cerebrospinal fluid (CSF). The bronchoalveolar lavage (BAL) sample was positive for influenza A.
Acyclovir	20 mg/kg/8 h IV	Day 1 to Day 3	Coverage for herpesviruses (HSV/VZV).	Discontinuation upon negative PCR HSV/VZV in CSF.
Vancomycin + Ceftriaxone Scheme	-Vancomycin: 15 mg/kg/8 h IV -Ceftriaxone: 100 mg/kg/day IV	10 days (from day 2 of hospitalization)	Broad coverage: Secondary bacterial meningitis initially suspected following neurological deterioration on day 2.	Persistent elevated inflammatory markers (CRP/PCT) and initial suspicion of bacterial coinfection
Immunoglobulin IV (IVIG)	1 g/kg/day IV	2 days (days 10–11 of hospitalization)	Immunological modulation: For H5N1-associated inflammatory/autoimmune encephalitis	Indicated by lack of neurological improvement (Glasgow 8/15) and persistent lesions on MRI.
Desmopressin	0.1 µg/12 h IV	6 days	Central diabetes insipidus: Correction of hypernatremia (Na 166 → 131 mEq/L).	Discontinuation upon normalization of urinary/serum osmolarity (Day 6).
Methylprednisolone	1 mg/kg/12 h IV (Day 5–7)	3 days	Anti-inflammatory: Trial for cerebral/CNS edema (partial response).	Not continued due to risk of viral replication.
Phenytoin	5 mg/kg/day IV	20 days	Anticonvulsant prophylaxis: For severe encephalitis.	
Oral Prednisone	1 mg/kg/day → weaning	Day 8	-Prevention of pulmonary/CNS inflammatory relapse.	

Abbreviations: PO, per os (oral); IV, intravenous; CSF, cerebrospinal fluid; BAL, bronchoalveolar lavage; HSV, herpes simplex virus; VZV, varicella-zoster virus; PCR, polymerase chain reaction; IVIG, intravenous immunoglobulin; CRP, C-reactive protein; PCT, procalcitonin; CNS, central nervous system; Na, sodium; mEq/L, milliequivalents per liter.

**Table 5:** Supporting interventions.

Intervention	Details	Duration	Effectiveness
Mechanical Ventilation	Protective parameters: PEEP 8 cmH_2_O, FiO_2_ 60%	Day 1–7	Improvement in PaO_2_/FiO_2_ ratio (200 → 350) Successful Extubation (Day 7)
Norepinephrine	0.1–0.3 µg/kg/min IV	Day 1–5	Septic Shock Management (MAP > 65 mmHg). Progressive Weaning (Day 5)
Early Rehabilitation	Respiratory + Motor Physiotherapy (Passive–Active)	Day 5–23	Partial Strength Recovery (Glasgow 9 → 15)
Enteral Nutrition	Nasogastric Tube (1200 kcal/day)	Day 3–23	Maintaining a Positive Nitrogen Balance

Abbreviations: PEEP, positive end-expiratory pressure; FiO_2_, fraction of inspired oxygen; PaO_2_, partial pressure of arterial oxygen; cmH_2_O, centimeters of water; MAP, mean arterial pressure; mmHg, millimeters of mercury.

## Data Availability

The original contributions presented in this study are included in the article. Further inquiries can be directed to the corresponding author.

## References

[1] World Health Organization (WHO) (2023). Cumulative Number of Confirmed Human Cases for Avian Influenza A(H5N1) Reported to WHO, 2003–2023. https://cdn.who.int/media/docs/default-source/influenza/h5n1-human-case-cumulative-table/cumulative-number-of-confirmed-human-cases-for-avian-influenza-a(h5n1)-reported-to-who--2003-2023071d9f0c-49c7-43ef-bf36-4ae01252b29a.pdf.

[2] World Organisation for Animal Health (WOAH) (2026). High Pathogenicity Avian Influenza (HPAI) Situation Report 78: Update as of 31 December 2025. https://www.woah.org/app/uploads/2026/01/hpai-situation-report-78.pdf.

[3] Imperia E., Bazzani L., Scarpa F., Borsetti A., Petrosillo N., Giovanetti M., Ciccozzi M. (2023). Avian influenza: Could the H5N1 virus be a potential next threat?. Microbiol. Res..

[4] Tripathi A.K., Sendor A.B., Sapra A. (2026). Avian Influenza. StatPearls.

[5] Zhang L., Liu K., Su Q., Chen X., Wang X., Li Q., Wang W., Mao X., Xu J., Zhou X. (2022). Clinical features of the first critical case of acute encephalitis caused by the avian influenza A (H5N6) virus. Emerg. Microbes Infect..

[6] National Emerging Special Pathogens Training and Education Center (NETEC) (2024). Pediatric Clinical Guidance on Highly Pathogenic Avian Influenza (HPAI) A(H5N1). https://netec.org/2024/12/17/pediatric-clinical-guidance-on-highly-pathogenic-avian-influenza-hpai-ah5n1/.

[7] Nakajima N., Van Tin N., Sato Y., Thach H.N., Katano H., Diep P.H., Kumasaka T., Thuy N.T., Hasegawa H., San L.T. (2013). Pathological study of archival lung tissues from five fatal cases of avian H5N1 influenza in Vietnam. Mod. Pathol..

[8] Centers for Disease Control and Prevention (CDC) (2025). Clinical Guidance for Evaluating Patients and Treatment and Post-Exposure Prophylaxis (PEP) of Influenza A(H5N1) Virus Infection. https://www.cdc.gov/bird-flu/hcp/clinicians-evaluating-patients/clinical-guidance-treatment.html.

[9] Uyeki T.M., Bernstein H.H., Bradley J.S., Englund J.A., File T.M., Fry A.M., Gravenstein S., Hayden F.G., Harper S.A., Hirshon J.M. (2019). Clinical Practice Guidelines by the Infectious Diseases Society of America: 2018 Update on Diagnosis, Treatment, Chemoprophylaxis, and Institutional Outbreak Management of Seasonal Influenza. Clin. Infect. Dis..

[10] Zhang X., Pu J., Sun Y., Bi Y., Jiang Z., Xu G., Zhang H., Cao J., Chang K.-C., Liu J. (2021). Neurovirulence of avian influenza virus is dependent on the interaction of viral NP protein with FMRP in the murine brain. J. Virol..

[11] Toovey S. (2008). Influenza-associated central nervous system dysfunction: A literature review. Travel Med. Infect. Dis..

[12] Armangue T., Moris G., Cantarín-Extremera V., Conde C.E., Rostasy K., Erro M.E., Portilla-Cuenca J.C., Turón-Viñas E., Málaga I., Muñoz-Cabello B. (2015). Autoimmune post–herpes simplex encephalitis of adults and teenagers. Neurology.

